# Cannabis Expectancies as a Mediator in the Relationship Between Cannabis Use and Psychiatric Symptoms in Women With Post-traumatic Stress Disorder (PTSD) Symptoms

**DOI:** 10.7759/cureus.108318

**Published:** 2026-05-05

**Authors:** Karina Villalba, Jennifer Attonito, Amie Newins

**Affiliations:** 1 Medicine, University of Central Florida, Orlando, USA; 2 Medical Education, Florida Atlantic University Charles E. Schmidt College of Medicine, Boca Raton, USA; 3 Psychology, University of Central Florida, Orlando, USA

**Keywords:** anxiety, cannabis, cannabis expectancies, depression, ptsd

## Abstract

Objective

This study examined whether cannabis expectancies mediate the relationship between lifetime cannabis use and symptoms of anxiety or depression among women who screened positive for post-traumatic stress disorder (PTSD).

Method

A total of 271 women completed an online survey assessing PTSD symptoms, lifetime cannabis use, anxiety and depression symptoms, and cannabis expectancies. Mediation analyses evaluated whether positive and negative cannabis expectancies explained the associations between lifetime cannabis use and anxiety or depression.

Results

The sample was predominantly White (238, 85%), with a mean age of 52.5 years (Mean±SD=12.2). Positive cannabis expectancies mediated the relationship between lifetime cannabis use and anxiety [indirect effect=0.35, Bootstrapped standard error of the effect estimate (BootSE)=0.15, 95% confidence interval (CI)=0.076 to 0.664] and depression (indirect effect=0.45, BootSE=0.19, 95% CI=0.12 to 0.88) such that women with longer cannabis use histories reported higher positive expectancies and higher levels of anxiety and depression.

Conclusions

Positive beliefs about cannabis’s anxiolytic and antidepressant effects may influence both the duration of use and psychological outcomes among women with PTSD. These findings highlight the role of expectancies in shaping the mental health impact of cannabis use in this population.

## Introduction

Cannabis continues to be the most widely consumed federally illegal substance in the U.S., and its use has escalated as Americans view it as less risky and more socially acceptable [1]. While men continue to use cannabis at higher rates, the gap between male and female users is closing, with more women now using cannabis to address issues like anxiety and depression [2]. This trend is reflected in recent national epidemiological data, showing a significant rise in cannabis use among women, from a prevalence of 2.6% in 2001 to 29.8% in 2016, as well as among pregnant and postpartum women, with a prevalence rate of 9.8% in 2020 [3, 4]. Research indicates that women use cannabis for mental health reasons such as anxiety (52%), depression (40%), and post-traumatic stress disorder (PTSD) (17%) [5]. This is further supported by data from a large U.S. registry, which shows that 14% of women seeking medical cannabis did so for mental health reasons, with 9% citing anxiety or PTSD as their primary condition [6].

Extensive research demonstrates that women are about twice as likely as men to develop PTSD following trauma exposure, with a lifetime prevalence of PTSD of approximately 10-12% in women and 5-6% in men [7]. In addition to the symptoms commonly associated with PTSD (i.e., avoidance, intrusive thoughts), women often experience anxiety and depression, significantly impacting their social functioning and overall quality of life [8]. While cross-sectional and prospective studies document the widespread use of cannabis by individuals with PTSD, evidence for cannabis’s effectiveness in managing PTSD, anxiety, or depression is limited [9, 10]. Some studies report short-term symptom relief, but systematic reviews and meta-analyses highlight insufficient evidence for long-term efficacy and caution against recommending cannabis for these conditions [9, 10].

Cannabis expectancies refer to the anticipated physical, cognitive, or behavioral changes that are believed to occur after cannabis consumption [11, 12]. Positive expectancies encompass effects such as relaxation or tension reduction and cognitive improvement. Alternatively, negative expectancies encompass anticipated adverse consequences of cannabis use, including increased cravings, appetite stimulation, and cognitive difficulties [12]. Recent studies indicate that a person’s beliefs in the potential of cannabis to reduce symptoms of anxiety and depression may contribute to their intention to use [13]. Those who hold positive expectations regarding the effects of cannabis products for reducing anxiety and/or depression are more likely to use it for a longer period or in larger quantities compared to those with less positive expectations [13]. Studies focusing on cannabis expectancies have found that positive expectancies regarding cannabis use are predictive of current and future use, while negative expectancies are associated with a lower likelihood of dependency and related problems [14].

While several studies have examined cannabis expectancies as mediators between cannabis use and mental health, no research to date has specifically focused on women with PTSD. This represents a significant gap in the literature and highlights the need for targeted research in this population. Among trauma‑exposed populations, more positive cannabis expectancies are associated with greater cannabis use and with mental health symptoms; however, these associations have not been examined specifically in women with PTSD [15]. We proposed to explore whether cannabis expectancies mediate the association between lifetime cannabis use and mental health symptoms among women with PTSD. We hypothesize that long-term cannabis use (i.e., more than three years) is associated with higher levels of positive cannabis expectancies. Furthermore, we predict that long-term cannabis use will be indirectly related to higher levels of anxiety and depression via its positive effect on cannabis-related expectancies.

## Materials and methods

The original sample for this cross-sectional study consisted of 412 individuals who reported cannabis use; however, only 271 participants reported PTSD (scored ≥3 on the PTSD-4 screener). If they scored less than or equal to 2 on the PTSD screener, they were excluded. Women were recruited via the Qualtrics online platform (Qualtrics LLC, Provo, USA) in 2022. We used Qualtrics to efficiently recruit a geographically diverse sample of women who use cannabis, a population that can be difficult to reach through traditional methods. However, because the sampling frame and recruitment algorithms of commercial panels are not fully transparent and participation is voluntary, our findings should be interpreted as applying to similar online, panel‑enrolled cannabis‑using women rather than all cannabis‑using women in the general population. Eligibility criteria included identifying as a woman, being 18 years or older, and self-reporting cannabis use in their lifetime. This study was approved by the University IRB. The continuous dependent variables included current (in the last 14 days) experience of anxiety and depression symptoms. The mediating continuous variables were positive and negative cannabis expectancies. The independent variable was lifetime cannabis use (0=<3 years and 1=≥3 years).

Measures

All the instruments are in the public domain and may be used without fee; please see the scales in Appendices 1-5.

PTSD

PTSD symptoms were screened with the 4‑item primary care PTSD screen (PC‑PTSD), a brief measure developed for use in medical settings that assesses re‑experiencing, avoidance, numbing, and hyperarousal. The PC‑PTSD has demonstrated adequate internal consistency, good diagnostic accuracy, and strong convergent validity with gold‑standard clinician interviews, supporting its use as a brief PTSD screener. A threshold of 3 maximized sensitivity of 0.78-0.85 and specificity of 0.82-0.87. Consistent with the original validation, we used a cutoff of ≥3 “yes” responses to identify probable PTSD cases [16].

Depression

The Patient Health Questionnaire-8 (PHQ-8) is a depression measure that can be used to assess current experiences of depression in population-based studies. It consists of eight items that assess the frequency of symptoms of depression, such as feeling down, loss of interest or pleasure, sleep problems, and changes in appetite or energy levels, over the past two weeks on a scale from 0 (not at all) to 3 (nearly every day). The total scores range from 0 to 24, with higher scores indicating more severe depressive symptoms. A cut-off score of 10 or higher is often used to define current depression [17].

Anxiety

The Overall Anxiety Severity and Impairment Scale (OASIS) answers a series of questions regarding their anxiety symptoms and how it affects their daily life. The scale consists of five questions that assess the severity and impairment associated with anxiety. Each question is scored on a scale from 0 to 4, with a total score range of 0 to 25. A total score of 5 or less represents mild anxiety, between 6 and 10 represents moderate anxiety, between 11 and 15 represents severe anxiety, and 16 or higher represents extreme anxiety. The OASIS is considered a valid and reliable measure for assessing anxiety severity and impairment in clinical settings [18].

Cannabis expectancies

The Cannabis Expectancy Questionnaire (CEQ) is a 60‑item measure used to assess the expectancies individuals have regarding the effects of cannabis use. It is designed to evaluate both positive and negative expectancies, covering aspects such as mood enhancement, relaxation, cognitive impairment, and negative emotional or physical consequences. Negative expectancies are typically associated with greater dependence severity, whereas positive expectancies are associated with higher consumption. Participants respond to statements on a 5‑point Likert scale ranging from 1=strongly disagree to 5=strongly agree. Following the original authors’ scoring procedure, positive and negative expectancy scores were computed by summing the respective items, yielding possible score ranges of 18 to 90 for positive expectancies and 27 to 135 for negative expectancies in the 45‑item version used here. Higher scores indicate stronger expectancies. In the current sample, internal consistency was excellent for both the positive (α=0.90) and negative (α=0.89) subscales [19].

Lifetime cannabis use

Participants were asked to report the number of years (in their lifetime) that they had used cannabis. Responses were dichotomized as 0=<3 years vs. 1=≥3 years. This categorization was selected based on clinical observations of cannabis use trajectories in trauma populations. We did not collect detailed information on cannabis potency, route of administration, or dose per episode, and we did not systematically assess current psychiatric medication or psychotherapy status in this study. Lifetime cannabis use was dichotomized as <3 years versus ≥3 years. This cutoff was selected to distinguish women with relatively recent or shorter‑term cannabis exposure from those with more sustained use, consistent with clinical observations that several years of regular cannabis use are often required for tolerance, dependence, and cannabis‑related problems to emerge, particularly in trauma‑exposed populations.

Analysis

Two mediation models were analyzed using the PROCESS macro Version 3 (University of Calgary, Calgary, Canada) for SPSS (IBM Corp., Armonk, USA). A total of four models were run to examine the indirect effect of lifetime cannabis use on anxiety/depression symptoms via cannabis expectancies. Separate models were run for each outcome variable (anxiety and depression symptoms) and for each type of cannabis expectancy (i.e., positive and negative), resulting in four models. All analyses were conducted using multiple ordinary least squares (OLS) regressions. Before interpreting the mediation models, we examined standard OLS regression assumptions, including linearity, homoscedasticity, normality, independence of residuals, and multicollinearity, to evaluate model fit and the potential impact of missing data. This method simultaneously estimates the direct association of X on Y (c’-path), the direct association of X on M (a-path), the direct association of M on Y (b-path), and the indirect association of X (lifetime cannabis use) on Y (anxiety and depression) via M (cannabis expectancies). The indirect effect (i.e., mediation) was tested using 10,000 resampling bias-corrected bootstrap confidence intervals (95% CI). The choice of OLS regression as the preferred method is justified, as it minimizes both type I and type II errors, offering greater power to detect mediational effects compared to similar approaches. Models were adjusted for race, ethnicity, education, and PTSD severity. Statistical computing was performed using SPSS V29.0.

## Results

A total of 271 women participated in this study. The mean age was 52.5 (Mean±SD=12.2) years old, and most participants identified as White and 7% as Hispanic. The majority of the participants were divorced, widowed, or separated 271 (46%). Most of them were college graduates, 271 (44%), and 44% were currently employed. The mean score for depression was 10.6 (Mean ±SD = 5.6), which indicated moderate symptoms, and the mean score for anxiety was 11.2 (Mean±SD=4.0), which also indicated moderate symptoms. Positive expectancy showed higher scores, showing more positive beliefs, 64.9 (Mean±SD=16.0), compared to negative beliefs, 50.0 (Mean±SD=10.7) (Table 1). The demographic information questions have been created by the authors; the Overall Anxiety Severity and Impairment Scale, measuring anxiety, the Patient Health Questionnaire, measuring depression, and the Cannabis Expectancy Scale are all freely available to the public. 

**Table 1:** Demographic characteristics PTSD: post-traumatic stress disorder.

Demographic Characteristics	PTSD
	n= 271
Age, mean (SD)	52.5 (12.2)
Race, No (%)	
White	238 (85%)
Black	31 (11%)
Multi-racial	12 (4%)
Hispanic, No (%)	
Yes	21 (7%)
No	260 (93%)
Marital Status, No (%)	
Single	37 (13%)
Living with a partner	31 (11%)
Married	85 (30%)
Separated, divorced, widow	128 (46%)
Education, No (%)	
Not a high school graduate	12 (4%)
High school graduate	59 (21%)
Some college	86 (31%)
College Graduate	124 (44%)
Employed No (%)	
Yes	123 (44%)
No	158 (56%)
Physical Health No (%)	
Good	170 (60%)
Fair	86 (31%)
Poor	25 (9%)
Mental Health mean (SD)	
Depression	10 (5.6)
Anxiety	11.2 (4.0)
Positive Expectancy	64.9 (16.0)
Negative Expectancy	50.0 (10.7)
Lifetime cannabis use No (%)	
2 years or less	108 (39%)
3 years or more	163 (61%)

Positive cannabis expectancies significantly mediated the relationship between lifetime cannabis use and both anxiety and depression.

**Figure 1:**
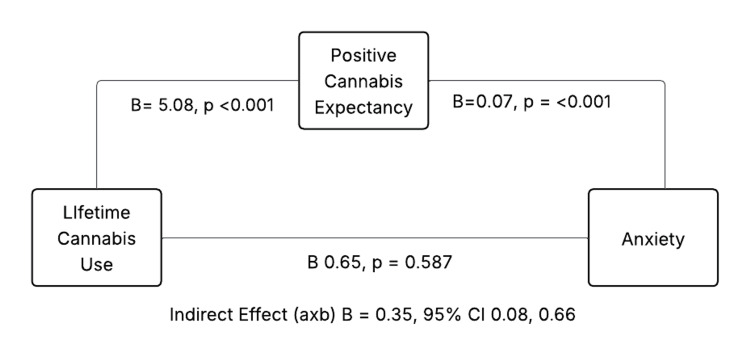
Indirect effect of positive cannabis use through lifetime cannabis use and anxiety among women with PTSD PTSD: post-traumatic stress disorder.

The indirect effect of positive cannabis use expectancies in the anxiety model was significant [indirect effect=0.35, BootSE=0.15, 95% confidence interval (CI)=0.08 to 0.66], whereas the direct effect of lifetime cannabis use on anxiety was non‑significant (B=0.65, SE=0.51, p=0.20), indicating full mediation (Figure 1, Tables 2, 3). The path for anxiety includes lifetime cannabis use → positive cannabis expectancy (B=5.08, p<0.001), positive cannabis expectancy → anxiety (B=0.07, p=0.005), and lifetime cannabis use → anxiety (B=0.65, p=0.20); the indirect effect (lifetime use → expectancies → anxiety) was B=0.35, 95% CI 0.08-0.66.

**Table 2:** Mediation anxiety model B: unstandardized regression coefficient, SE: standard error, CI: confidence interval, Bootstrap samples: 10,000, Confidence level: 95%.

Anxiety Model Path / Effect	B	SE	t	p	95% CI
a path: Lifetime Cannabis Use → Pos Cannabis Expectancies	5.08	1.24	4.09	<0.001	[2.64, 7.53]
b path: Pos Cannabis Expectancies → Anxiety	0.07	0.02	2.85	< 0.001	[0.02, 0.12]
c’ path (direct effect): Lifetime Cannabis Use → Anxiety	0.65	0.51	1.28	0.202	[-0.35, 0.64]
Indirect effect (a × b): Lifetime Cannabis Use → Pos Cannabis Expectancies → Anxiety	0.35	0.15	—	—	[0.08, 0.66]

**Table 3:** Mediation anxiety model B: unstandardized regression coefficient, SE: standard error, CI: confidence interval, Bootstrap samples: 5000, Confidence level: 95%.

Model	Anxiety Model Dependent Variable	R	R²	F(df₁, df₂)	p
1	Pos Cannabis Expectancies (Mediator)	0.19	0.04	22.95 (1, 271)	< 0.01
2	Anxiety (Outcome)	0.16	0.07	14.79 (2, 271)	< 0.001

**Figure 2:**
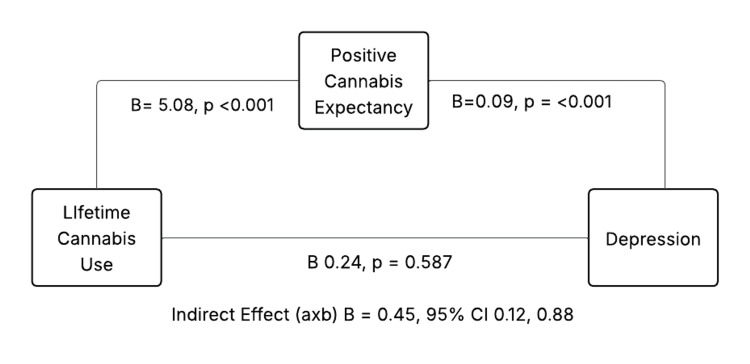
Indirect effect of positive cannabis use through lifetime cannabis use and depression among women with PTSD PTSD: post-traumatic stress disorder.

The indirect effect of positive cannabis expectancies in the depression model was also significant (indirect effect=0.45, BootSE=0.19, 95% CI=0.12 to 0.88) among women with PTSD, whereas the direct association between lifetime cannabis use and depression was not significant (B=0.24, SE=0.70, p=0.73), indicating full mediation (Figure 2, Tables 4, 5). The path for depression includes lifetime cannabis use → positive cannabis expectancy (B = 5.08, p <0.001), positive cannabis expectancy → depression (B=0.09, p=0.007), and lifetime cannabis use → depression (B=0.24, p=0.73); the indirect effect (lifetime use → expectancies → depression) was B=0.45, 95% CI 0.12-0.88. 

**Table 4:** Mediation depression model B: unstandardized regression coefficient, SE: standard error, CI: confidence interval.

Depression Model Path / Effect	B	SE	t	p	95% CI
a path: Lifetime Cannabis Use → Pos Cannabis Expectancies	5.08	1.24	4.09	< 0.001	[2.64, 7.53]
b path: Pos Cannabis Expectancies → Depression	0.09	0.03	2.71	0.007	[0.04, 0.15]
c′ path (direct effect): Lifetime Cannabis Use → Depression	0.24	0.70	-0.35	0.728	[−0.61, 1.34]
Indirect effect (a × b): Lifetime Cannabis Use → Pos Cannabis Expectancies → Depression	0.45	0.19	—	—	[0.12, 0.88]

**Table 5:** Mediation depression model B: unstandardized regression coefficient, SE: standard error, CI: confidence interval, PHQ: Patient Health Questionnaire score; LIFE_M: life meaning, PosEXP: positive experience.

Model	Depression Model Dependent Variable	R	R²	F (df₁, df₂)	p
1	Positive Exposure (Mediator)	0.24	0.06	16.76 (1, 271)	< .001
2	Depression (Outcome)	0.16	.03	3.73 (2, 270)	0.0252

In the models for negative expectancies, there was no significant indirect effect of negative expectancies on either anxiety or depression.

The depression model was not statistically significant, R²=0.01, F(1,271)=3.51, p=0.062. Lifetime marijuana use was not significantly associated with negative expectancies, b=-3.81, SE=2.04, p=0.062, 95% CI [-7.82, 0.19]. Lifetime marijuana use was significantly directly associated with depression, b=-1.56, SE=0.76, p=0.043, 95% CI [-3.06, -0.05], whereas negative expectancies were not significantly associated with depression, b=0.00, SE=0.02, p=0.90, 95% CI [-0.04, 0.05]. The indirect effect of lifetime marijuana use on depression through negative expectancies was not statistically significant, b=-0.01, bootstrapped SE=0.10, 95% bootstrap CI [-0.22, 0.21], indicating no evidence of mediation.

The anxiety model was also not statistically significant, R²=0.01, F(1, 271)=3.51, p=0.062. Lifetime marijuana use was not significantly associated with negative expectancies, b=-3.81, SE=2.04, p=0.062, 95% CI [-7.82, 0.19]. Neither lifetime marijuana use [b=-0.42, SE=0.58, p=0.47, 95% CI (-1.55, 0.72)] nor negative expectancies [b=-0.01, SE=0.02, p=0.72, 95% CI (-0.04, 0.03)] showed a significant association with anxiety symptoms. The indirect effect of lifetime marijuana use on anxiety through negative expectancies was not statistically significant, b=0.02, bootstrapped SE=0.09, 95% bootstrap CI [-0.14,0.22], indicating no evidence of mediation.

## Discussion

The present study examined the mediating role of positive and negative cannabis use expectancies in the relationship between lifetime cannabis use and current anxiety and depressive symptoms among women with PTSD. Consistent with expectancy theory, participants with greater lifetime exposure reported stronger positive effect expectancies, which in turn were associated with more severe anxiety and depression. 

The finding of the indirect associations for both anxiety and depression indicates that, in this group, lifetime cannabis exposure is linked to current internalizing symptoms largely through the belief that cannabis relieves negative affect, rather than through a direct association with symptom severity. These results support expectancy theory as a cognitive mechanism for self‑medication. If an individual expects a substance to relieve negative affect, this expectancy increases the likelihood of use and helps maintain use over time [20-22]. The Catastrophizing, Anxiety, Negative Urgency, and Expectancy (CANUE) framework for understanding substance use identified expectancies as modifiable risk factors in the pathway from distress to substance use [23, 24]. Similarly, we identified positive expectancies in the pathway between lifetime cannabis use and mental health. 

While our cross‑sectional mediation findings are consistent with broader evidence linking cannabis use and internalizing symptoms, longitudinal work indicates that the directionality is complex and likely bidirectional, varying by population, outcome, and pattern of use. Longitudinal evidence suggests small and often bidirectional associations between cannabis use and anxiety, whereby cannabis use can precede higher anxiety in some cohorts, and pre-existing anxiety can increase the likelihood of cannabis use, including for self-medication [25, 26]. Our finding that positive cannabis use expectancies were indirectly associated with lifetime cannabis use and both depressive and anxiety symptoms supports this literature by highlighting cognitive coping processes rather than direct pharmacologic effects as proximal mechanisms linking cannabis exposure with internalizing symptoms in women with PTSD [27-30]. However, because cannabis use, expectancies, and symptoms were measured at a single time point, temporal ordering cannot be established. It is not possible to determine whether cannabis use led to the development of stronger positive expectancies and higher symptoms, whether pre-existing symptoms and expectancies increased the likelihood of cannabis use, or whether bidirectional processes were operating. In addition, our study did not measure cannabis frequency, dose, and concurrent psychiatric treatment, which may confound the observed associations and limit causal inference, especially with respect to self‑medication and symptom severity. Future studies should incorporate more granular cannabis use metrics (e.g., frequency, potency, and mode of use) and psychiatric treatment indicators and use designs capable of modeling time‑varying confounding (e.g., longitudinal cohorts). Finally, dichotomizing lifetime cannabis use may reduce sensitivity to dose-response relationships; future work should treat cannabis use duration as a continuous or multi‑category variable when possible.

Findings from this investigation should be interpreted in light of several limitations. First, although validated self-report measures were used, such questionnaires are subject to recall and social desirability bias, which can affect the accuracy of reported cannabis use and related behaviors. Second, the cross-sectional design limits the ability to draw causal inferences or fully assess mediating effects. However, because cannabis use (the independent variable) was collected retrospectively and mediators and outcomes were assessed based on current experiences, some temporal ordering is established, lending partial support to the study design. Third, the models may not account for all possible mediators, as unmeasured variables could influence the observed relationships. Fourth, the sample consisted primarily of White, non-Hispanic women, which restricts the generalizability of findings to other racial and ethnic groups. Finally, based on cannabis expectancy theory, a bidirectional relationship between cannabis use and mental health outcomes may exist, further complicating interpretation. Despite these limitations, the study provides important theoretical contributions and lays the groundwork for future longitudinal mediation analyses with more diverse and representative samples.

## Conclusions

Despite these limitations, this study offers clinically relevant insights by delineating the role of positive cannabis expectancies among women with PTSD. The findings suggest that interventions should target not only the behavioral pattern of cannabis use but also the maladaptive cognitions that sustain it. Specifically, cognitive‑behavioral strategies that challenge the belief that cannabis is the only effective option for symptom relief may help reduce problematic use and improve mental health outcomes. Future research employing longitudinal designs with more diverse clinical samples is needed to clarify the bidirectional nature of these associations and to inform intervention development.

## Appendices

Appendix 1

**Table 6:** Expectancy table CEQ: Questions and response options were available in the validation study published article [19]. The scale is publicly available on [31].

#	Statement 1 = Strongly Disagree, 2= Disagree, 3= Neither Agree nor disagree, 4= Agree, 5 = Strongly Agree	1	2	3	4	5
1	I get better ideas when using marijuana	☐	☐	☐	☐	☐
2	Little things annoy me less when I am using marijuana	☐	☐	☐	☐	☐
3	I am more worried about what others are saying about me when I am using marijuana	☐	☐	☐	☐	☐
4	Using marijuana makes me feel outgoing and friendly	☐	☐	☐	☐	☐
5	Using marijuana makes me feel tense	☐	☐	☐	☐	☐
6	I have more self-confidence when using marijuana	☐	☐	☐	☐	☐
7	I have bizarre or strange thoughts when using marijuana	☐	☐	☐	☐	☐
8	I use marijuana to get full enjoyment out of life	☐	☐	☐	☐	☐
9	Using marijuana makes me more sexually responsive	☐	☐	☐	☐	☐
10	Using marijuana makes me confused	☐	☐	☐	☐	☐
11	I am more aware of what I say and do when I am using marijuana	☐	☐	☐	☐	☐
12	I feel restless when using marijuana	☐	☐	☐	☐	☐
13	I am more depressed when using marijuana	☐	☐	☐	☐	☐
14	Using marijuana makes me feel sluggish	☐	☐	☐	☐	☐
15	When I use marijuana, I withdraw from others	☐	☐	☐	☐	☐
16	When I use marijuana, it is easier to express my feelings	☐	☐	☐	☐	☐
17	Using marijuana increases my tension	☐	☐	☐	☐	☐
18	When I use marijuana, I find it hard to get certain thoughts out of my head	☐	☐	☐	☐	☐
19	When I use marijuana, I feel less motivated	☐	☐	☐	☐	☐
20	Using marijuana makes me laugh	☐	☐	☐	☐	☐
21	I tend to adopt a “who cares” attitude when using marijuana	☐	☐	☐	☐	☐
22	Using marijuana makes me more easily irritated	☐	☐	☐	☐	☐
23	I feel less shy if I have been using marijuana	☐	☐	☐	☐	☐
24	Using marijuana helps me to feel “normal” again	☐	☐	☐	☐	☐
25	When I use marijuana, my mood feels flat	☐	☐	☐	☐	☐
26	Using marijuana makes me happy	☐	☐	☐	☐	☐
27	Using marijuana helps me concentrate	☐	☐	☐	☐	☐
28	When I am using marijuana, I avoid people or situations for fear of embarrassment	☐	☐	☐	☐	☐
29	When I use marijuana, I can speak my mind	☐	☐	☐	☐	☐
30	I am disappointed in myself when using marijuana	☐	☐	☐	☐	☐
31	I tend to avoid sex if I have been using marijuana	☐	☐	☐	☐	☐
32	I am clumsier when using marijuana	☐	☐	☐	☐	☐
33	Marijuana helps me to get along with others	☐	☐	☐	☐	☐
34	Using marijuana makes me feel insecure	☐	☐	☐	☐	☐
35	When using marijuana, I do things that I do not really mean to do	☐	☐	☐	☐	☐
36	Using marijuana gives me more energy	☐	☐	☐	☐	☐
37	When using marijuana people find it difficult to understand me	☐	☐	☐	☐	☐
38	I have more energy when using marijuana	☐	☐	☐	☐	☐
39	I lose most feelings of sexual interest after I have been using marijuana	☐	☐	☐	☐	☐
40	Marijuana makes me feel jumpier and more agitated	☐	☐	☐	☐	☐
41	When I use marijuana, I feel “panicky”	☐	☐	☐	☐	☐
42	Using marijuana makes me feel excited	☐	☐	☐	☐	☐
43	When I use marijuana, I have thoughts that are not my own	☐	☐	☐	☐	☐
44	When using marijuana my feelings shift rapidly from one to another	☐	☐	☐	☐	☐
45	When using marijuana, I feel out of touch with reality	☐	☐	☐	☐	☐

Appendix 2 

**Table 7:** PTSD screener This measure was developed by staff at VA's National Center for PTSD and is in the public domain and not copyrighted [32]. PTSD: post-traumatic stress disorder.

In your life, have you ever had any experience that was so frightening, horrible, or upsetting that, in the past 12 months you	Yes	No
Have had nightmares about it or thought about it when you did not want to?		
Tried hard not to think about it or went out of your way to avoid situations that reminded you of it?		
Were constantly on guard, watchful, or easily startled?		
Felt numb or detached from others, activities, or your surroundings?		

 Appendix 3

**Table 8:** Demographic characteristics Credits: Karina Villalba PhD and Jennifer Attonito PhD.

Q#	Item / Question	Response Type
1	What is your age?	Open-ended (numeric)
2	Are you of Hispanic/Latino origin or descent?	Single choice: Yes / No
2a	Which of the following best describes your Hispanic/Latino origin?	Single choice: Spaniard from Spain, Mexican, Central American, South American, Puerto Rican, Cuban, Dominican, Caribbean, Haitian, Other (describe), Don't know, Refused
3	What is your race?	Single choice: White, Black/African American, Caribbean/West Indian, Haitian/Haitian American, Asian, Multi-racial (describe), Other (describe), Don't know, Refused
4	What was your sex at birth?	Single choice: Male / Female
5	What is your current gender identity?	Single choice: Male, Female, Transgender, Other (describe), Don't know, Refused
6	What is your sexual orientation?	Single choice: Heterosexual/Straight, Gay/Lesbian, Bisexual, Asexual, Other (specify)
7	What is your current relationship status?	Single choice: Married, Divorced, Widowed, Separated, Never Married/Single, Living with long-term partner
8	What is the highest grade or year of school you completed?	Single choice: Elementary or below, Some high school, High school graduate/GED, Some college/technical, College/trade graduate, Graduate/professional degree
9	Are you currently employed?	Single choice: Yes / No
10	Which of the following best describes your current status?	Single choice: Employed for wages, Self-employed, Retired, Unemployed, Unable to work, Disabled, Other
11	During the past 12 months, have you had any kind of health insurance or health coverage?	Single choice: Yes / No
11a	Please check all the types of health insurance you had during the past 12 months.	Multiple choice: Private, Medicaid, Medicare, Ryan White HIV/AIDS Program, VA Coverage, Jackson Card, Obamacare (ACA), Other (describe)
12	Overall, how would you rate your health in the past month?	Single choice: Excellent, Very good, Good, Fair, Poor, Very poor

Appendix 4 

**Table 9:** Depression scale Depressive symptoms were assessed using the Patient Health Questionnaire (PHQ‑8), an 8‑item self‑report measure of depression severity [16]. The scale can be freely downloaded at [33].

	Not at all (0)	Several days (1)	Over half the days (2)	Nearly every day (3)
Little interest or pleasure in doing things?				
Feeling down, depressed or hopeless				
Trouble falling asleep, staying asleep, or sleeping too much				
Feeling tired or having little energy				
Poor appetite or overeating				
Feeling bad about yourself - or that you’re a failure or have let yourself or your family down				
Trouble concentrating on things, such as reading the newspaper or watching television				
Moving or speaking so slowly that other people could have noticed. Or, the opposite - being so restless that you have been moving around a lot more than usual				

Appendix 5 

**Table 10:** Anxiety scale This scale is publicly available at [34].

	Not at all	Mild	Moderate	Severe	Extreme
How often do you feel anxious?					
When you feel anxious, how intense or severe is your anxiety?					
H ow often do you avoid situations, places, objects, or activities because of anxiety or fear?					
How much does anxiety or fear interfere with your ability to do the things you need to do at work, at school, or at home?					
How much does anxiety or fear interfere with your social life and relationships?					
Feeling afraid, as if something awful might happen					
